# Systematic combinatorial optimization of three-phage cocktails against multidrug-resistant *Pseudomonas aeruginosa*

**DOI:** 10.1128/spectrum.04096-25

**Published:** 2026-06-15

**Authors:** Meshack Tweya Omwega, Moses Gachoya, Justin Nyasinga, Martin Georges, Collins Kigen, Kevin Kamanyi, Samuel Nyamweya, Erick Odoyo, Lillian Musila

**Affiliations:** 1 Kenya Medical Research Institute, Kericho, Kenya; 2 Department of Applied Health Sciences, Kisii University 361787, Kisii, Kenya; 3 Microbiology Hub Kericho, Walter Reed Army Institute of Research–Africa 107864, Kericho, Kenya; 4 Department of Medical Biochemistry and Parasitology, Kisii University 217802, Kisii, Kenya; 5 Department of Pathology, Egerton University, Nakuru, Kenya; University of California San Diego, La Jolla, California, USA

**Keywords:** bacteriophage therapy, *Pseudomonas aeruginosa*, antimicrobial resistance, phage cocktails, phage interactions, synergy, biofilm, Highest Single Agent independence model, combinatorial optimization, *Galleria mellonella*, invertebrate infection model, phage cocktail design

## Abstract

Multidrug-resistant (MDR) *Pseudomonas aeruginosa* poses a significant global health threat, necessitating alternative therapeutic strategies. Bacteriophage therapy shows promise, but single-phage use is prone to resistance. Bacteriophage cocktails can address this issue, but they are typically formulated empirically without a systematic evaluation of constituent phage interactions. We employed a comprehensive combinatorial approach to optimize three-phage cocktails against clinical *P. aeruginosa* isolates. From 25 candidate bacteriophages, 5 were selected based on a broad host range (≥60% of 51 MDR *P. aeruginosa* clinical isolates). Ten possible three-phage cocktail combinations were systematically generated and evaluated using real-time Omnilog phenotypic microarray analysis. Phage interactions were quantified using the Highest Single Agent independence model to classify synergistic (Δ > +5%), neutral (−5% ≤ Δ ≤ +5%), or antagonistic (Δ < −5%) effects. Individual phage inhibition efficiencies ranged from 35.4% to 75.4%. Cocktail performance varied dramatically (16.1%–84.1% inhibition). Only one cocktail exhibited synergy, achieving 84.1% inhibition, an 8.7% improvement over the best individual phage. Four cocktails (40%) showed neutral interactions, while five (50%) demonstrated antagonistic effects. The optimal cocktail (Phages 1 + 3 + 4) demonstrated efficacy across six of eight diverse clinical isolates in biofilm inhibition assays (29%–63% biomass reduction, 2.7–3.6 log_10_ colony-forming unit reduction) and achieved 80% survival in *Galleria mellonella* versus 10% in infected controls. These findings demonstrate that phage cocktail optimization requires rational validation, as antagonistic interactions can occur, establishing a critical validation step before committing resources to *in vivo* efficacy studies.

**IMPORTANCE:**

Bacteriophage cocktails are increasingly proposed as alternatives to antibiotics against multidrug-resistant bacteria, yet their development remains largely empirical. This study challenges the assumption that the combination of effective phages yields additive or synergistic benefits. Through systematic evaluation of all possible three-phage combinations from five phages, we discovered that antagonistic interactions (50%) predominated over synergistic effects (10%), with some cocktails performing worse than individual phages. Using the Highest Single Agent independence model, we established a quantitative framework for rational cocktail design that identified a therapeutically superior formulation. This approach is generalizable across bacterial pathogens and provides a critical validation step for phage therapy development. Our findings demonstrate that rational testing of phage interactions is essential before *in vivo* or clinical translation, potentially preventing costly failures and accelerating the development of effective phage-based therapeutics against multidrug-resistant infections.

## INTRODUCTION

*Pseudomonas aeruginosa* remains a leading cause of difficult-to-treat nosocomial infections, particularly affecting immunocompromised individuals, burn patients, and those with cystic fibrosis due to extensive antibiotic resistance mechanisms (1, 2). The World Health Organization identifies carbapenem-resistant *P. aeruginosa* as a critical priority pathogen, with multidrug-resistant bloodstream infections resulting in mortality rates exceeding 40% (3, 4). The escalating antibiotic resistance crisis necessitates alternative therapeutic strategies, with bacteriophage therapy emerging as a promising approach offering high bacterial specificity (5). However, phage therapy faces limitations, including narrow host ranges and rapid development of bacterial resistance (6).

Phage cocktails, combinations of multiple bacteriophages with complementary lytic properties, have been proposed to broaden bacterial coverage and reduce resistance emergence through multi-target activity (7). Despite their therapeutic potential, phage cocktail development remains largely empirical, with limited understanding of the interactions among constituent phages. These interactions may be synergistic or additive, enhancing overall efficacy beyond individual components or antagonistic (reducing combined efficacy below expectations), yet systematic frameworks for evaluating these phenomena are not widely available or adopted (8). The absence of standardized methodologies for quantifying phage interactions and comparing cocktail performance hinders the systematic combinatorial optimization of phage therapeutic formulations (9).

Beyond rational cocktail design, *in vivo* validation of the phage cocktails needs an infection model that is physiologically relevant and practically feasible. The *Galleria mellonella* (greater wax moth larvae) has become a useful intermediate system that can be used to bridge the gap between *in vitro* and mammalian models on the effects of antimicrobials in the presence of bacterial pathogens such as *P. aeruginosa* (10, 11). This invertebrate model has an innate immune system that mimics mammalian immunity, both cellular (hemocytes) and humoral (antimicrobial peptides) responses, and infection can be induced at human physiological temperature (37°C), and it provides ethical and economic advantages over vertebrate models while maintaining predictive value for therapeutic outcomes (12, 13). *G. mellonella* has been successfully used to assess both bacterial virulence and phage therapeutic efficacy, making it appropriate to evaluate the efficacy of optimized phage cocktails.

This study addresses the critical gaps in systematic combinatorial optimization of three-phage cocktails against *P. aeruginosa*. We employed high-throughput Omnilog phenotypic microarray analysis to screen a bacteriophage panel, generated all possible three-phage combinations, and quantified phage interactions using the Highest Single Agent (HSA) independence model. Multi-criteria decision analysis identified the optimal therapeutic cocktail, which was validated across genetically diverse clinical isolates through biofilm inhibition assays and *G. mellonella* infection models. This evidence-based approach establishes a reproducible experimental framework for systematic combinatorial optimization of phage cocktails, providing a data-driven platform for improving the therapeutic efficacy of phage-based interventions against multidrug-resistant *P. aeruginosa* infections.

## MATERIALS AND METHODS

### Bacterial strains and culture conditions

Fifty-one de-identified clinical isolates of multidrug-resistant *P. aeruginosa* from patients with wound swabs from different geographical locations across Kenya were obtained from an ongoing antimicrobial surveillance program at the Microbiology Hub, Kericho WRAIR, Kenya (Table S1). All the isolates were classified as multidrug-resistant (defined as resistance to at least one agent in ≥3 antimicrobial categories) and represented different strain types. Strain PA01, susceptible to all the selected phages, served as the representative test strain for cocktail evaluation. All bacterial cultures were grown in tryptic soy broth (TSB) at 37°C with orbital shaking at 180 rpm. Overnight cultures were diluted to approximately 1 × 10⁷ colony-forming units (CFUs)/mL in fresh medium for experimental inoculation. Long-term cultures were preserved at −80°C in TSB supplemented with 20% glycerol.

### Bacteriophage isolation and characterization

#### Phage selection and efficiency of plating quantification

Twenty-five lytic bacteriophages targeting *P. aeruginosa* were previously isolated from wastewater samples collected in Kenya (14). Efficiency of plating (EOP) was calculated as the ratio of the phage titer for each test clinical isolate to that for the reference isolation host. For each phage-bacteria combination, serial 10-fold dilutions (10⁻¹–10⁻⁸) of phage lysate were prepared in SM buffer (100 mM NaCl, 8 mM MgSO_4_, 50 mM Tris-HCl [pH 7.5], and 0.01% gelatin) and plated using the double-layer agar technique with 0.7% top agar. Median EOPs were computed across all 51 isolates for each phage.

#### Host range determination

Host range profiling was conducted through spot assays (15). Briefly, 5 μL aliquots of each phage lysate (standardized to 10^9^ PFU/mL) were spotted onto bacterial lawns of all 51 clinical isolates prepared using the double-layer agar overlay technique. Plates were incubated at 37°C for 18–24 h, and lysis zones were visually assessed. The phages with a broad host range, defined as productive infection (EOP ≥0.1) against ≥60% of the 51 clinical *P. aeruginosa* isolates, were then selected. Five bacteriophages met the selection criterion (productive infection against ≥60% of clinical isolates) and advanced to combinatorial analysis: vB_PaePA01phi1_RS1 (Phage 1), vB_PaePA10145phi1_RS1 (Phage 2), vB_PaePA8132phi1_PS3 (Phage 3), vB_PaePA10145phi1_HR2 (Phage 4), and vB_PaePA10145phi1_MKR2 (Phage 5).

#### Phage propagation and titer standardization

High-titer phage stocks were prepared using the plate lysate method (15). Phages were propagated on their respective isolation host strains using the double-layer agar technique. Crude lysates were harvested by flooding plates with SM buffer, incubating at 4°C overnight, and collecting supernatant. Lysates were clarified by centrifugation at 10,000 × *g* for 10 min at 4°C and filtered through 0.22 μm filters. Phage titers were determined by standard double-layer agar plaque assay and expressed as PFU/mL. All phage stocks were standardized to 1 × 10^9^ PFU/mL in SM buffer for equimolar cocktail formulation. Titers were reverified immediately before each experiment.

#### Whole-genome sequencing

Phage DNA was extracted from the high-titer lysates of the five bacteriophages (>10^9^ PFU/mL) using the QIAamp Mini Kit (Qiagen, Germantown, MD, USA) following the manufacturer’s protocol. DNA concentration and purity were assessed using a Nanodrop One Spectrophotometer and a Qubit fluorometer (Thermo Fisher Scientific Inc., Waltham, MA, USA). Whole-genome sequencing was performed using Illumina NextSeq 1000 (Illumina Inc., San Diego, CA, USA) with a reagent kit (300 cycles, 2 × 150 bp reads). Raw reads were quality-filtered and trimmed using BBDuk v38.18, and *de novo* assembly was performed using SPAdes v4.0.0. Assembly quality was assessed using QUAST v5.3.0.

#### Functional annotation and safety screening

Complete genome sequences of the five selected bacteriophages were analyzed for undesirable genetic elements that would preclude therapeutic use. Draft genomes were annotated using Pharokka v1.7.4 (16), MMseqs2 v13.45111 (17), and Phanotate v1.6.6 (18–20). Phage lifestyle was predicted using PhageLeads (https://phageleads.dk/) (21). Using Pharokka annotation, phage genomes were screened for undesirable genetic elements by BLAST against the Comprehensive Antibiotic Resistance Database, version 3.2.4 (accessed on 15 November 2025), the Virulence Factor Database, version 6.0 (accessed on 15 November 2025), and custom lysogeny marker databases. No genes encoding integrases, repressors, antimicrobial resistance determinants, or known toxins were identified in any of the five phages.

#### Genomic diversity and taxonomic classification

Whole-genome sequences of the five selected bacteriophages were analyzed using the Virus Intergenomic Distance Calculator (VIRIDIC). VIRIDIC calculates pairwise intergenomic similarities based on genomic sequence alignment, providing standardized viral taxonomy aligned with the International Committee on Taxonomy of Viruses criteria (22). Complete phage genomes were submitted in FASTA format with default parameters: minimum alignment length, 500 bp; BLAST E-value threshold, 1 × 10^−5^; and alignment coverage filtering. Taxonomic relationships were inferred based on established thresholds: phages sharing ≥95% intergenomic similarity were considered the same species, those sharing 70%–95% similarity were classified as the same genus but different species, and phages with <70% similarity were assigned to different genera (23).

### Cocktail formulation

#### Combinatorial generation of three-phage cocktails

Three-phage cocktail combinations were systematically generated using combinatorial mathematics. Three-phage cocktails were selected based on three converging rationales. First, three-component formulations have historical precedent in successful phage therapy products and recent clinical applications (24, 25). Second, with five candidate phages, C (5,3) = 10 unique three-component cocktails represent a fully tractable experimental set; evaluating two-phage combinations [C (5,2) = 10] sacrifices coverage breadth, whereas four-phage combinations [C (5,4) = 5] lose combinatorial resolution. Third, prior work has shown that three-phage combinations provide efficacy advantages over single phages while minimizing the antagonism risk associated with larger cocktails (25–27). The number of unique combinations was calculated using the binomial coefficient formula. This yielded 10 distinct combinations designated: A (1 + 2 + 3), B (1 + 2 + 4), C (1 + 2 + 5), D (1 + 3 + 4), E (1 + 3 + 5), F (1 + 4 + 5), G (2 + 3 + 4), H (2 + 3 + 5), I (2 + 4 + 5), and J (3 + 4 + 5).

Each cocktail was prepared immediately before experimental application by mixing equal volumes of three constituent phage suspensions, each standardized to 1 × 10^9^ PFU/mL.

#### Modified Appelmans protocol for cocktail formulation integrated with the Omnilog system

The Omnilog phenotypic microarray system (Biolog Inc., Hayward, CA, USA) was employed for real-time, high-throughput quantitative assessment of bacterial growth dynamics and phage-mediated growth inhibition. This platform monitors bacterial metabolic activity through tetrazolium redox dye reduction (Biolog Redox Dye Mix A), producing a colorimetric signal proportional to cellular respiration and NADH production (28).

Bacterial culture (*P. aeruginosa* PA01) in the mid-exponential phase (OD_600_ = 0.4–0.6) was diluted to 1 × 10^7^ CFU/mL in TSB supplemented with 2 mM CaCl_2_ and 1× Biolog Redox Dye Mix A. One hundred microliters of the bacterial suspension was dispensed into individual wells of Omnilog microplates (Biolog PM-M plates). Phage preparations (individual phages or cocktails) were added at a multiplicity of infection (MOI) = 10, while control wells received equivalent volumes of sterile SM buffer. Three control conditions were included: (i) positive control (bacteria without phage), (ii) negative control (sterile TSB with dye but no bacteria), and (iii) phage-only control (phage without bacteria). Microplates were sealed and immediately transferred to the Omnilog reader and incubated at 37°C with continuous kinetic monitoring. Colorimetric readings were recorded every 15 min over 24 h, generating comprehensive growth kinetic profiles.

#### Quantification of antibacterial activity using area under the curve metrics

Calorimetric data were acquired using Biolog’s Data Analysis Software version 1.7. Kinetic profile data were analyzed using GraphPad Prism. Area under the curve (AUC) was calculated for each growth kinetic profile as the primary metric quantifying total cumulative bacterial metabolic activity over the 24-h monitoring period. AUC values were computed by numerical integration using the trapezoidal rule, where *y_i_* represents colorimetric measurement (Omnilog units) at time point *i*, *t_i_* represents the corresponding time in hours, and *n* represents the total measurement time points.

To enable standardized comparison, inhibition efficiency (IE) was calculated:


$$IE\;\left(\%\right)=\frac{\left(AUC_{control}-\;AUC_{treatment}\right)}{\left(AUC_{control}-\;AUC_{negative}\right)}\;\times \;100,$$

where AUC_control represents the area under the curve for untreated bacterial growth, AUC_treatment represents the area for phage-treated cultures, and AUC_negative represents the baseline signal from sterile media. This calculation provides an intuitive interpretation, where 0% indicates no inhibition, and 100% indicates complete inhibition.

#### Highest Single-Agent independence model for interaction classification

The HSA independence model (29) was applied to quantify phage interactions within cocktails. For three-component combinations, expected inhibition efficiency under independence was calculated as:


$$IE_{expected}\left(\%\right)=\;Max\left({IE}^{1},\;{IE}^{2},\;{IE}^{3}\right),$$

where IE₁, IE₂, and IE₃ represent individual inhibition efficiencies of three constituent phages. The interaction parameter (Δ) quantifying deviation from independence prediction was calculated as:


Δ (%) = IE_observed − IE_expected,

where IE_observed represents experimentally measured inhibition efficiency of the complete cocktail.

Cocktails were classified into three categories based on Δ values: (i) synergistic: Δ > +5% (observed performance significantly exceeds best individual phage, *P* < 0.05), (ii) neutral: −5% ≤ Δ ≤ +5% (observed performance approximates best individual phage), and (iii) antagonistic: Δ < −5% (observed performance significantly falls below best individual phage, *P* < 0.05).

#### Bioinformatic analysis of biofilm-associated genes

The biofilm-forming potential of the clinical isolates was assessed by analyzing whole-genome sequences of the strains for biofilm-associated genes using Abricate against the Virulence Factor Database using the following parameters: minimum DNA sequence identity, 75%; minimum coverage, 50%; and BLAST E-value cutoff, 1 × 10⁻⁵. Target biofilm-related virulence factors included quorum-sensing regulators (*lasI, lasR, rhlI,* and *rhlR*), exopolysaccharide (EPS) synthesis operons (*pelA-G, pslA-D,* and *alg* genes), c-di-GMP signaling components (*wspF, yfiN,* and *roeA*), type IV pili genes (*pilA-W*), flagellar biosynthesis genes (*fliC and flgK*), and biofilm-regulatory genes (*mucA, mucB,* and *mucD*). Gene presence/absence patterns were visualized using the R package pheatmap (version 4.4.2) with hierarchical clustering. Eight phenotypically diverse clinical isolates (MTPA05, MTPA22, MTPA30, MTPA34, MTPA35, MTPA45, MTPA46, and MTPA50) were selected from the original 51-strain panel based on their genetically defined biofilm-forming capacity.

#### Biofilm formation and quantification

The biofilm disruption activity of the selected cocktail was tested on the reference strain PA01 and the eight clinical isolates with biofilm potential. Biofilm assays were conducted in 48-well flat-bottom polystyrene microplates following crystal violet staining protocols. Overnight bacterial cultures were diluted to OD_600_ = 0.1 (approximately 1 × 10⁹ CFU/mL) in fresh TSB supplemented with 2% glucose. Three hundred ninety-six microliters of TSB was added to each well, followed by 10 μL of bacterial suspension. Plates were incubated statically at 37°C for 24 h. After biofilm maturation, the phage cocktail was added at an MOI of 10 and incubated for another 24 h. Planktonic cells were removed by aspiration, and wells were washed three times with 400 μL sterile phosphate-buffered saline (PBS, pH 7.4). Biofilms were fixed with 1 mL of methanol, dried for 15 min, and stained with 1 mL of 1% crystal violet solution for 15 min. Excess stain was removed by washing five times with distilled water, followed by air-drying. Crystal violet bound to biofilm biomass was solubilized using 1 mL of 33% acetic acid with gentle agitation for 15 min. Absorbance was measured at 595 nm (OD_595_) using a microplate spectrophotometer (SpectraMax M2^e^).

#### Viable cell enumeration from biofilms

Following 24-h biofilm formation and planktonic cell removal, biofilms were scraped using sterile cell scrapers and resuspended in 400 μL PBS by vigorous vortexing for 2 min. Serial 10-fold dilutions (10^−1^–10^−8^) were prepared and plated onto tryptic soy agar using the spread-plate technique. Plates were incubated at 37°C for 18–24 h, and colonies were enumerated to determine the CFU/mL. Biofilms were dispersed by scraping with sterile cell scrapers, followed by vigorous vortexing for 2 min in 400 μL PBS. This method may not achieve complete single-cell disaggregation; CFU counts should therefore be interpreted as an operational measure of viable units rather than absolute single-cell enumeration.

#### Optimization of the *G. mellonella* infection model

*G. mellonella* larvae provide an invertebrate infection model with an innate immune system analogous to mammals, enabling assessment of *in vivo* therapeutic efficacy without the ethical constraints of vertebrate models (11). The model was optimized to establish a reproducible *P. aeruginosa* infection.

Healthy *G. mellonella* larvae (250–350 mg body weight) were selected and stored in darkness at 15°C. Before selection, larvae were propagated and maintained following standard *G. mellonella* husbandry protocols (30). After propagation, larvae with uniform pigmentation, no melanization spots, and vigorous movement were selected 24 h before infection. Ten larvae per treatment group were used.

A mid-exponential-phase bacterial culture (OD_600_ = 0.5) of strain PA01 was serially diluted to obtain concentrations ranging from 10¹ to 10⁵ CFU per 10 μL inoculum. Groups of 10 larvae were infected via injection into the left proleg using a 10 μL Hamilton syringe fitted with a 26-gauge needle. Control groups received PBS only. Larvae were incubated at 37°C in darkness and monitored for survival over 72 h. Death was defined as the complete absence of movement in response to gentle tactile stimulation and melanization. The LD_50_ was calculated using linear interpolation (Finney’s method) between the two doses, bracketing 50% mortality.

Observed mortality rates were 10^1^ CFU (40%), 10^2^ CFU (50%), 10^3^ CFU (80%), 10^4^ CFU (70%), 10^5^ CFU (100%), and PBS control (0%). Calculated LD_50_ was 10^1.5^ CFU/larva (approximately 32 CFU per larva).

#### Bacterial infection and phage treatment to test *in vivo* efficacy in the *G. mellonella* model

To evaluate the efficacy of the optimal cocktail, an *in vivo* survival assay was performed using the optimized *G. mellonella* infection model. *P. aeruginosa* strain PA01 was cultured to the mid-exponential phase (OD_600_ = 0.5), pelleted by centrifugation (5,000 × *g*, 10 min), and washed twice in sterile PBS. Bacterial inoculum was verified by serial dilution plating to confirm 10^1.5^ CFU per 10 μL injection volume. Larvae were infected via injection into the left proleg. Each larva received 10 μL of bacterial suspension (1 × 10^1.5^ CFU per larva). Phage treatment was administered 1 h post-infection via injection into the right proleg. Treatment groups received 10 μL of cocktail to achieve an MOI of 10. An MOI of 10 was selected based on a preliminary optimization study. Control groups included: (i) infected control (bacteria + SM buffer), (ii) phage-only control (phage without bacteria), (iii) uninfected control (PBS injection only), and (iv) untreated control (no injection). Following treatment, larvae were incubated in sterile petri dishes lined with filter paper at 37°C in darkness.

#### Survival monitoring and health assessment

Larval survival and health status were monitored every 12 h for 72 h post-infection. Three physiological parameters were assessed: (i) activity score (0–4 scale: 0, no movement; 1, minimal movement when touched; 2, movement of head/body segments when touched; 3, slow movement without touch; and 4, active movement); (ii) melanization score (0–4 scale: 0, black larvae; 1, black spots on brown larvae; 2, >3 melanization spots; 3, <3 melanization spots; and 4, no melanization); and (iii) cocoon formation [scored as present (1), partial (0.5), or absent (0)]. All assessments were performed by two independent observers blinded to treatment groups. Median scores were calculated for each group at each time point.

### Statistical analysis

All experiments included three independent biological replicates. Continuous ratio-scale data are presented as mean ± standard deviation (SD). Coefficient of variation (CV) was calculated as (SD/mean) × 100. Normality was assessed using the Shapiro-Wilk test; one-way analysis of variance (ANOVA) tested for significant differences among cocktail formulations, with normality assessed using the Shapiro-Wilk test. *Post hoc* pairwise comparisons employed Tukey’s honest significant difference test. One-sample *t* tests compared observed cocktail inhibition efficiencies against HSA-predicted values. For biofilm assays, Wilcoxon signed-rank tests were used to compared treated versus untreated conditions. For *G. mellonella* survival analysis, Kaplan-Meier survival curves were generated and compared using the log-rank test. Kruskal-Wallis tests with *post hoc* Dunn’s tests were used to analyze activity, melanization scores, and cocoon formation rates, with results reported as median (interquartile range [IQR]). Statistical significance was defined as *P* < 0.05. All analyses were performed using R statistical software (version 4.4.2).

## RESULTS

### Bacteriophage isolation and characterization

#### Selection and characterization of bacteriophages

Systematic screening of 25 bacteriophage isolates identified candidates with therapeutic potential against *Pseudomonas aeruginosa*. The selection criterion required productive infection (EOP ≥0.1) against at least 60% of the 51 clinical isolates. Five bacteriophages met this criterion: vB_PaePA01phi1_RS1 (Phage 1), vB_PaePA10145phi1_RS1 (Phage 2), vB_PaePA8132phi1_PS3 (Phage 3), vB_PaePA10145phi1_HR2 (Phage 4), and vB_PaePA10145phi1_MKR2 (Phage 5). These phages demonstrated productive infection against 60.8%–86.3% of clinical isolates tested (Table 1), making them the most effective candidates from the initial screening panel.

**TABLE 1:** Characteristics of selected bacteriophages against *P. aeruginosa* clinical isolates^*a*^

Phage	Productive infections	Median EOP	Mean EOP ± SD
Phage 1 (RS1)	41/51 (80.4%)	0.66	0.61 ± 0.38
Phage 2 (RS1-2)	43/51 (84.3%)	0.80	0.75 ± 0.42
Phage 3 (PS3)	37/51 (72.5%)	0.64	0.60 ± 0.45
Phage 4 (HR2)	31/51 (60.8%)	0.71	0.60 ± 0.55
Phage 5 (MKR2)	44/51 (86.3%)	0.60	0.64 ± 0.43

^*a*^ EOP, efficiency of plating; IQR, interquartile range; and SD, standard deviation. *n* = 51 clinical isolates tested per phage.

Host range matrix analysis revealed complementary lytic profiles among selected phages (Fig. S1). Hierarchical clustering identified three distinct groups based on host specificity patterns. The first cluster comprised Phages 2 (RS1-2) and 5 (MKR2), characterized by highly similar infection profiles and consistently strong activity across a broad range of isolates. The second cluster included Phages 3 (PS3) and 4 (HR2), demonstrating more selective but efficient lysis patterns. Phage 1 (RS1) formed a distinct third cluster, exhibiting a unique host specificity profile that successfully lysed several isolates resistant to other phages. Notably, one clinical isolate (MT-PA-44) demonstrated complete resistance to all five selected phages.

#### Genomic diversity and taxonomic classification

**Fig 1:**
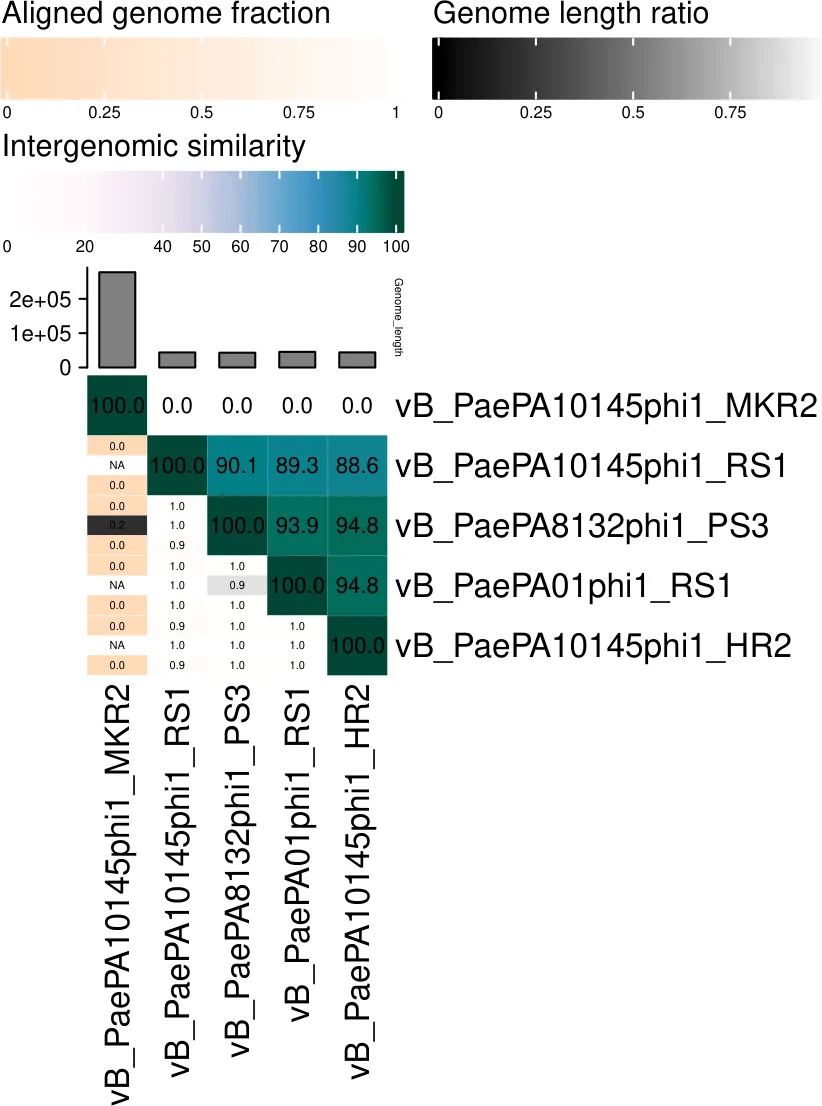
Phylogenetic relationships among selected bacteriophages based on whole-genome sequence analysis determined by VIRIDIC.

VIRIDIC analysis revealed intergenomic similarities ranging from 0.0% to 94.8% among the five selected bacteriophages (Fig. 1). The panel comprised representatives from two distinct viral genera. The first genus, *Phikmvirus* (*n* = 4, Phage 1 [vB_PaePA01phi1_RS1], Phage 2 [vB_PaePA10145phi1_RS1], Phage 3 [vB_PaePA8132phi1_PS3], and Phage 4 [vB_PaePA10145phi1_HR2]), belongs to the group *Myophages*. Pairwise similarities within this cluster were as follows: Phage 1-2 (89.3%), Phage 1-3 (93.9%), Phage 1-4 (94.8%), Phage 2-3 (90.1%), Phage 2-4 (88.6%), and Phage 3-4 (94.8%). All values fell below the 95% species threshold, classifying these phages as members of the same genus but distinct species according to ICTV criteria. The second genus was *Phikzvirus* (*n* = 1) (Phage 5 [vB_PaePA10145phi1_MKR2]), which exhibited no sequence similarity to all other phages, with no detectable sequence alignment (aligned genome fraction = 0.0%), with other phages in public databases. The absence of homologous genomic regions suggests that this phage represents a phylogenetically distant lineage.

### Cocktail formulation

#### Individual phage performance assessment

Each of the five selected phages was individually evaluated against PA01 using the modified Appelmans protocol for cocktail formulation with continuous Omnilog monitoring. Individual phage inhibition efficiencies ranged from 35.4% to 75.4% (Table 2). Phage 4 demonstrated the highest antibacterial activity with an inhibition efficiency of 75.4% ± 0.6% and an AUC of 1,331.4 ± 30.0. This superior performance represented a 35.2% improvement over the second-best phage (Phage 2, 40.2%). The remaining four phages exhibited moderate and remarkably similar inhibition efficiencies, differing by less than 5 percentage points. Phage 2 (40.2% ± 1.1%, AUC 3,234.9 ± 60.8), Phage 3 (38.2% ± 1.6%, AUC 3,343.9 ± 87.6), Phage 5 (38.0% ± 1.0%, AUC 3,355.0 ± 55.8), and Phage 1 (35.4% ± 2.3%, AUC 3,498.6 ± 124.0).

**TABLE 2:** Individual phage performance metrics^*a*^

Phage	AUC (mean ± SD)	Inhibition efficiency (%)	Inhibition time (h)	CV (%)	Rank
Phage 1	3,498.6 ± 124.0	35.4 ± 2.3	11.5 ± 0.7	6.5	5
Phage 2	3,234.9 ± 60.8	40.2 ± 1.1	11.5 ± 0.7	2.7	2
Phage 3	3,343.9 ± 87.6	38.2 ± 1.6	11.0 ± 0.0	4.2	3
Phage 4	1,331.4 ± 30.0	75.4 ± 0.6	21.0 ± 1.4	0.8	1
Phage 5	3,355.0 ± 55.8	38.0 ± 1.0	12.0 ± 0.0	2.6	4

^*a*^ Controls: positive (bacteria only) AUC = 5,411.6 ± 50.5; negative (media only) AUC = 1,275.1 ± 48.5; phage only (no bacteria) AUC = 1,289.3 ± 52.1. CV, coefficient of variation; AUC, area under the curve. All experiments were performed using three independent biological replicates.

#### Systematic evaluation of all 10 three-phage cocktail combinations

**Fig 2:**
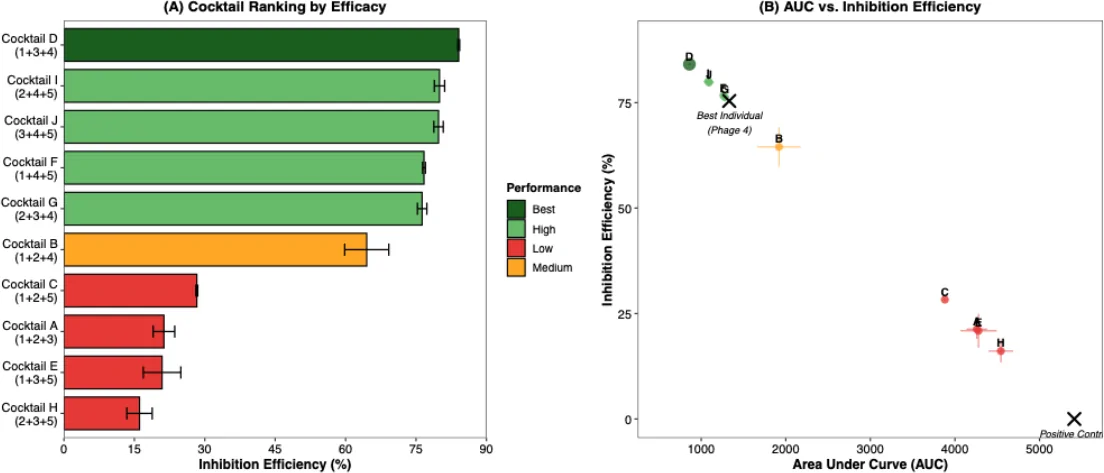
Comparative performance analysis of three-phage cocktail combinations. (**A**) Ranked bar plot showing inhibition efficiency (%) for all 10 cocktail combinations. Bars are color-coded by performance tier: green (high performance, >75%), orange (intermediate performance, 60%–75%), and red (low performance, <30%). Error bars represent standard deviation (*n* = 3 biological replicates). Dark green bar indicates best individual phage performance (Phage 4, 75.4%). Statistical comparison by one-way ANOVA followed by *post hoc* Tukey HSD; Shapiro-Wilk normality test results are referenced in Table S4. (**B**) Scatter plot showing the relationship between area under the curve and inhibition efficiency for all cocktails. Lower AUC values correspond to greater bacterial growth inhibition. Cocktails are color-coded as in panel A.

Cocktail inhibition efficiencies ranged from 16.1% to 84.1% (Table S2), representing a 5.2-fold performance difference. One-way ANOVA revealed significant differences among cocktail formulations (F_9,20_ = 422.6, *P* = 9.78 × 10⁻¹³). *Post hoc* Tukey Honestly Significant Difference (HSD) testing identified three statistically distinct performance tiers (Fig. 2A).

High-performance tier (>75% inhibition): five cocktails exceeded the performance of the best individual phage (Phage 4, 75.4%): Cocktail D (84.1% ± 0.2%), I (80.0% ± 1.1%), J (79.8% ± 1.0%), F (76.7% ± 0.3%), and G (76.3% ± 1.0%). These formulations demonstrated statistically superior efficacy compared to all other groups (*P* < 0.001). Intermediate-performance tier (60%–70% inhibition): Cocktail B achieved 64.5% ± 4.7% inhibition, significantly lower than high-performance cocktails (*P* < 0.001) but superior to low-performance formulations (*P* < 0.001). Low-performance tier (<30% inhibition): four cocktails exhibited severely diminished efficacy: C (28.3% ± 0.2%), A (21.3% ± 2.3%), E (20.9% ± 4.0%), and H (16.1% ± 2.7%). These values fell below the least effective individual phage (Phage 1, 35.4%), indicating antagonistic interactions among constituent phages.

Analysis of constituent phage composition revealed performance patterns (Fig. 2B; Fig. S2). All five high-performance cocktails contained Phage 4, whereas four of the five low-performance cocktails excluded Phage 4. Additionally, four low-performance cocktails contained either a combination of Phages 1 + 2 (Cocktails A, B, and C) or Phages 2 + 3 + 5 (Cocktail H). Notably, Cocktail I, despite containing Phages 2 + 5, achieved high performance through the inclusion of Phage 4, suggesting Phage 4 may buffer antagonistic effects.

Cocktail D (Phages 1 + 3 + 4) emerged as the optimal formulation, achieving 84.1% ± 0.2% inhibition efficiency with superior reproducibility (CV = 1.52%, lowest among all formulations). This represented an 8.7 percentage point improvement over the best individual phage and a 48.7 percentage point improvement over the poorest individual phage.

#### HSA independence analysis

The HSA independence model was applied to all 10 phage cocktails to quantify the nature of interactions among constituent phages. Δ values ranged from −24.2% to +8.7% (Table 3), revealing diverse interaction outcomes. Only Cocktail D (Phages 1 + 3 + 4) exhibited synergistic interaction (Δ = +8.7%, 95% CI: +7.9%, +9.5%, *P* < 0.001), achieving 84.1% inhibition. Four cocktails (I, J, F, and G) displayed neutral interactions (Δ within ±5%). Five cocktails (B, C, E, A, and H) demonstrated antagonistic interactions, with Cocktail H (Phages 2 + 3 + 5) showing the most pronounced antagonism (Δ = −24.2%, 95% CI: −28.4%, −20.0%). Interaction classification frequencies: synergistic 10% (1/10), neutral 40% (4/10), and antagonistic 50% (5/10).

**TABLE 3:** HSA independence analysis results for three-phage cocktails^*a*^

Cocktail	Phages	Observed IE (%)	Expected IE (%)	Δ (%)	95% CI	Interaction type	*P*-value
D	1 + 3 + 4	84.1 ± 0.2	75.4	+8.7	+7.9,+9.5	Synergistic	<0.001
I	2 + 4 + 5	80.0 ± 1.1	75.4	+4.6	+3.1,+6.1	Neutral	0.003
J	3 + 4 + 5	79.8 ± 1.0	75.4	+4.4	+2.9,+5.9	Neutral	0.006
F	1 + 4 + 5	76.7 ± 0.3	75.4	+1.3	+0.9,+1.7	Neutral	0.009
G	2 + 3 + 4	76.3 ± 1.0	75.4	+0.9	−0.7,+2.5	Neutral	0.242
B	1 + 2 + 4	64.5 ± 4.7	75.4	−10.9	−15.6,−6.2	Antagonistic	<0.001
C	1 + 2 + 5	28.3 ± 0.2	40.2	−11.9	−12.4,−11.4	Antagonistic	<0.001
E	1 + 3 + 5	20.9 ± 4.0	38.2	−17.3	−21.2,−13.4	Antagonistic	<0.001
A	1 + 2 + 3	21.3 ± 2.3	40.2	−18.9	−21.2,−16.6	Antagonistic	<0.001
H	2 + 3 + 5	16.1 ± 2.7	40.2	−24.2	−28.4,−20.0	Antagonistic	<0.001

^*a*^ IE, inhibition efficiency; Δ, interaction parameter (observed − expected). Expected IE was determined by the HSA model (maximum IE among constituent phages). Interaction classification: synergistic (Δ > +5%), neutral (−5% ≤ Δ ≤ +5%), antagonistic (Δ < −5%). 95% confidence intervals for Δ were computed from one-sample *t* tests*. P* values from one-sample *t* tests comparing observed IE to expected IE; *n* = 3 biological replicates.

Among tested cocktails, only one cocktail (D; Phages 1 + 3 + 4) exhibited synergistic interaction, achieving 84.1% inhibition, which exceeded the performance of the best single phage (Phage 4, 75.4%) by 8.7 percentage points (*P* < 0.001). Cocktail D is the only combination in which the mixed formulation provided a measurable therapeutic benefit beyond the most effective individual phage.

Four cocktails (I, J, F, and G) displayed neutral or additive interactions, with Δ values within ±5% threshold. Their inhibition efficiencies approximated or slightly exceeded that of the most active single phage. Five cocktails (B, C, E, A, and H) demonstrated antagonistic interactions (Δ < −5%), exhibiting inhibition levels markedly below HSA expectations. Moderate antagonism was observed for cocktails B and C. Severe antagonism was evident in cocktails E, A, and H. Most pronounced antagonism occurred in Cocktail H (Phages 2 + 3 + 5), which achieved only 16.1% inhibition, equivalent to 40% of its kexpected HSA value (40.2%).

Overall, interaction classification frequencies were as follows: synergistic, 10% (1/10); neutral, 40% (4/10); and antagonistic, 50% (5/10) (Fig. S3C). Mean interaction parameter differed markedly among classes, averaging +8.7% for synergistic, +2.8% ± 0.9% for neutral, and −16.6% ± 2.7% for antagonistic combinations.

### Multi-strain biofilm inhibition assays

#### Biofilm disruption of multiple clinical isolates by the optimal phage cocktail

To validate the therapeutic potential of Cocktail D beyond the single screening strain PA01, biofilm inhibition assays were conducted against eight phenotypically diverse clinical isolates representing the genetic heterogeneity of the clinical isolates. Whole-genome sequencing and bioinformatics analysis characterized the genetic potential for biofilm formation in clinical isolates, enabling the rational selection of eight strains representing diverse biofilm mechanisms, including PA01 (Fig. S4).

#### Biofilm biomass reduction

**Fig 3:**
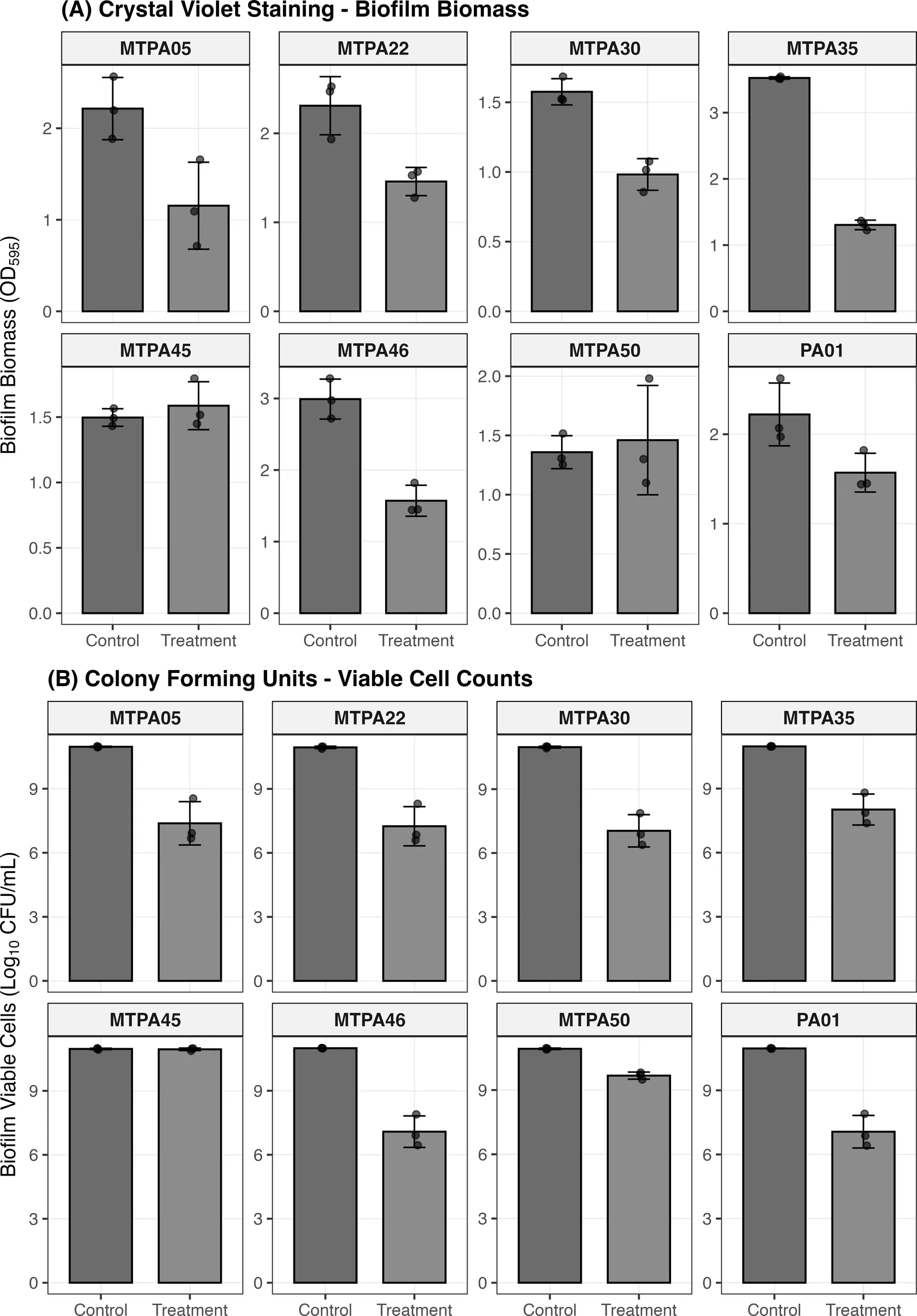
Multi-strain biofilm inhibition by Cocktail D. (**A**) Bar graphs showing crystal violet-quantified biofilm biomass (OD_595_) for untreated and Cocktail D-treated conditions across eight strains. Error bars represent standard deviation (*n* = 3). (**B**) Bar graphs showing viable cell counts (log_10_ CFU/mL) from biofilms for untreated and treated conditions. Error bars represent standard deviation (*n* = 3). The graphs were generated by R statistical software (version 4.4.2).

Crystal violet staining revealed substantial inhibition of biofilm formation across six of the eight *P. aeruginosa* strains following phage cocktail treatment (Fig. 3A; Table S3). Untreated control biofilms showed OD_595_ values ranging from 1.36 to 3.52 (mean 2.21 ± 0.75), indicating robust biofilm establishment.

Following Cocktail D treatment, six strains exhibited significant biomass reductions (Wilcoxon signed-rank test *P* < 0.05). MTPA35 exhibited the greatest susceptibility with 63% biomass reduction, followed by MTPA05 (48% reduction), MTPA46 (47% reduction), MTPA30 (38% reduction), MTPA22 (37% reduction), and PAO1 (29% reduction). Two strains demonstrated resistance to Cocktail D. MTPA45 and MTPA50 showed no significant biomass reduction.

#### Viable cell enumeration from biofilms

CFU enumeration corroborated the crystal violet results, demonstrating marked reductions in viable biofilm-associated cells across all responsive strains (Fig. 3B; Table S3). Control biofilms exhibited mean bacterial densities between 10.93 and 10.99 log_10_ CFU/mL. Cocktail D treatment reduced viable cell counts by 2.95–3.93 log_10_ in the six responsive strains. The greatest CFU reductions were observed in MTPA30 (3.93 log_10_ reduction, *P* < 0.05), MTPA46 (3.91 log_10_ reduction, *P* < 0.01), PAO1 (3.90 log_10_ reduction, *P* < 0.05), MTPA22 (3.70 log_10_ reduction, *P* < 0.05), MTPA05 (3.58 log_10_ reduction, *P* < 0.05), and MTPA35 (2.95 log_10_ reduction, *P* < 0.01). These reductions confirm potent disruption activity within established biofilms. Consistent with biomass measurements, MTPA45 displayed minimal CFU reduction (0.02 log_10_), confirming genuine resistance. MTPA50 showed intermediate reduction (1.26 log_10_), suggesting partial susceptibility insufficient for therapeutic efficacy.

### *In vivo* efficacy validation

#### *In vivo* efficacy assessment in the *G. mellonella* infection model

**Fig 4:**
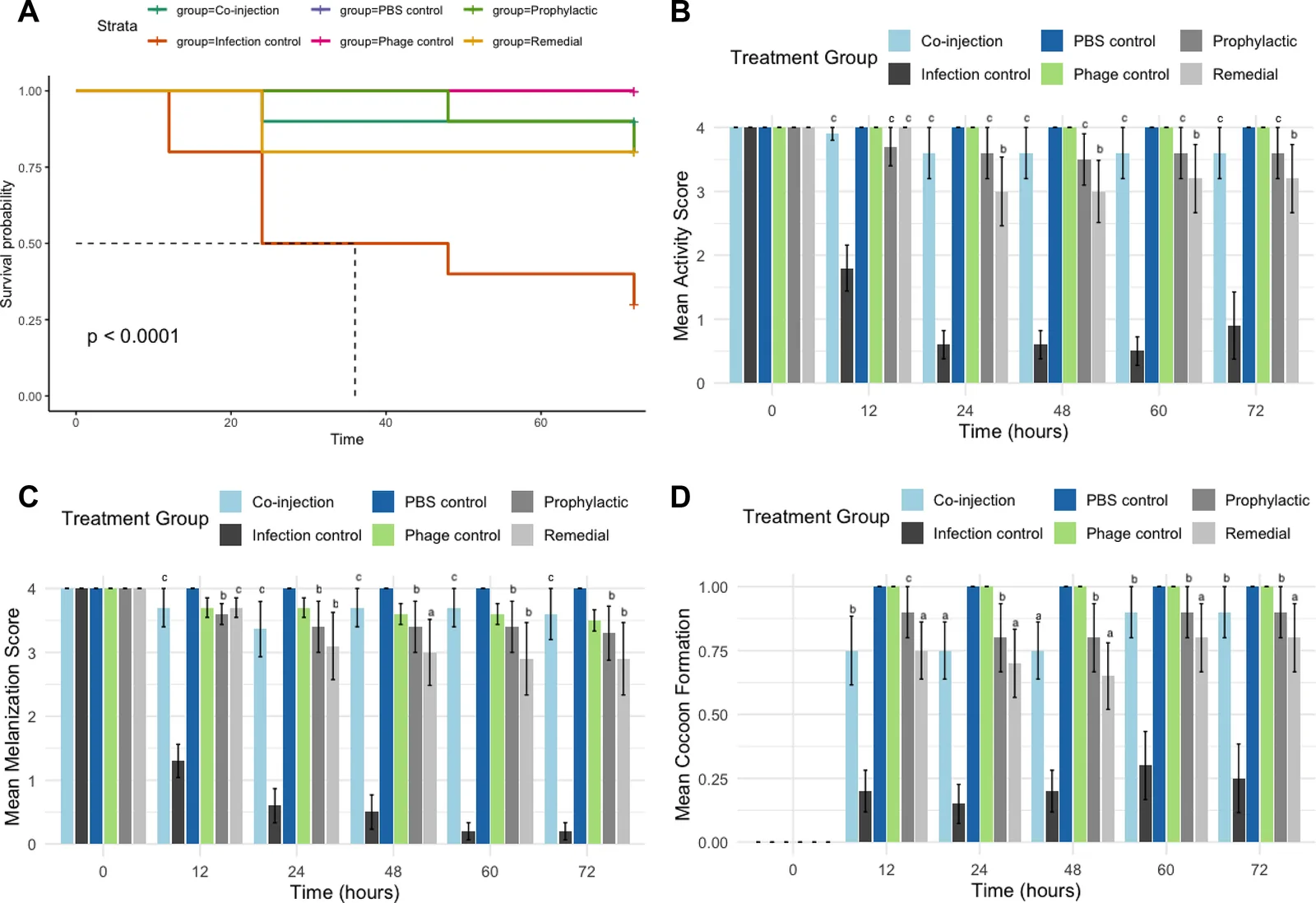
*In vivo* efficacy assessment in *G. mellonella* infection model. (**A**) Kaplan-Meier survival curves of *G. mellonella* larvae infected with *P. aeruginosa* MRSN PA01. Treatment groups: co-injection (bacteria + phage), PBS control, prophylactic (phage before bacteria), infection control (bacteria only), phage control (phage only), and remedial (phage after bacteria). Log-rank test: *P* < 0.0001. *n* = 10 larvae per group. Hazard ratios with 95% CIs from the Cox proportional hazards model are reported in Table S4. (**B**) Activity scores (0–4 scale) over time. Bars represent median (IQR). Kruskal-Wallis tests: significant at all time points (*P* < 0.001). (**C**) Melanization scores (0–4 scale) over time. Higher scores indicate no melanization. Bars represent median (IQR). Kruskal-Wallis tests: significant at all time points. (**D**) Cocoon formation rates at 72 h post-infection, showing the percentage of larvae forming cocoons. Bars represent median (IQR). Kruskal-Wallis tests: significant at all time points. a, *P* < 0.05; b, *P* < 0.01; and c, *P* < 0.001 (Dunn’s *post hoc* tests comparing phage-treated groups to infection control).

To evaluate therapeutic efficacy in a physiologically relevant system, phage Cocktail D was tested in *G. mellonella* larvae infected with *P. aeruginosa* PA01. The *G. mellonella* model allows assessment of phage efficacy in a living host with functional immune responses while avoiding the complexity and ethical considerations of mammalian models. Survival analysis using Kaplan-Meier survival curves revealed dramatic therapeutic efficacy (Fig. 4A). Infected control larvae (bacteria only) exhibited rapid mortality onset at approximately 20 h, with survival declining to 50% by approximately 32 h and reaching approximately 30% cumulative survival by 72 h. In contrast, phage-treated larvae demonstrated markedly improved survival: co-injection and prophylactic treatment maintained approximately 85% survival at 72 h, and remedial treatment (phage treatment after 1 h of bacterial challenge) maintained approximately 80% survival throughout the observation period. The phage control and PBS control groups maintained 100% survival throughout the study. Log-rank test confirmed highly significant survival differences between infected controls and phage-treated groups (*P* < 0.0001). Hazard ratios with 95% confidence intervals (CIs) for each phage-treated group vs infected control are reported in Table S5.

#### Activity score dynamics

Activity assessments revealed sustained health in phage-treated larvae (Fig. 4B). All groups maintained median activity scores of 4.0 at baseline (0 h), indicating normal healthy larvae with active movement. Phage-treated groups maintained high activity scores throughout all time points from 12 to 72 h. In contrast, infected controls showed a dramatic decline: scores dropped to approximately 1.8 ± 0.5 at 12 h, then declined further to approximately 0.5 ± 0.3 at 24 h, remained at approximately 0.5 ± 0.3 at 48 h, increased slightly to approximately 0.6 ± 0.3 at 60 h, and rose to approximately 0.8 ± 0.4 at 72 h. This pattern indicates severe morbidity with minimal movement throughout most of the observation period, with slight recovery in surviving larvae at later time points. Kruskal-Wallis tests revealed significant treatment effects at all time points. *Post hoc* Dunn’s tests confirmed significant differences between phage-treated and infected controls at all time points (*P* < 0.001)

#### Melanization response

Melanization scoring monitored changes in larval pigmentation across treatment groups (Fig. 4C). All groups began with melanization scores of 4.0 ± 0.0 at baseline (0 h), representing healthy beige larvae with no melanization. Infected control larvae exhibited progressive loss of pigmentation and decline in melanization scores: dropping from 4.0 at 0 h to approximately 1.3 at 12 h, 0.5 at 24 h, 0.5 at 48 h, and 0.3 at 72 h, predominantly reflecting accumulation of melanin due to overwhelming infection. Phage-treated larvae maintained significantly higher melanization scores throughout the study, indicating preserved normal larval and minimal infection. Kruskal-Wallis tests confirmed significant differences among treatment groups at all time points. Dunn’s *post hoc* tests revealed that the phage-treated groups differed significantly from the infected controls at all time points (Fig. 4C).

#### Cocoon formation as a health indicator

Cocoon formation served as an integrative health endpoint assessed over time post-infection (Fig. 4D). At baseline (0 h), no larvae had formed cocoons across all groups (median cocoon formation = 0.00). By 12 h, phage-treated larvae began developing cocoons with high formation rates: co-injection (approximately 0.90), PBS control (approximately 1.00), prophylactic (approximately 0.85), phage control (approximately 1.00), and remedial (approximately 0.90). These elevated rates were maintained throughout the observation period, with all phage-treated groups showing cocoon formation values of approximately 0.95–1.00 at 24, 48, 60, and 72 h, indicating normal physiological functions and developmental progression. In stark contrast, infected control larvae showed dramatically impaired cocoon formation: approximately 0.25 at 12 h, then declining to approximately 0.10–0.15 at 24 h, and remaining at approximately 0.20–0.30 from 48 to 72 h, consistent with high mortality and severe morbidity in this group. Kruskal-Wallis tests confirmed significant differences among treatment groups at all time points from 12 h onwards. Dunn’s *post hoc* tests revealed significantly higher cocoon formation in all phage-treated groups compared to infected controls at all time points (Fig. 4D).

## DISCUSSION

This systematic combinatorial evaluation optimized three-phage cocktails against *P. aeruginosa* and revealed a predominance of antagonistic interactions (50%) over synergistic effects (10%), contradicting expectations of additive benefits from random phage combinations. This finding aligns with recent observations that phage-antibiotic combinations can exhibit antagonistic interactions in multidrug-resistant bacterial strains, including *P. aeruginosa*, with protein synthesis inhibitors inducing antagonism in some cases (31). This suggests that negative interactions extend beyond phage-antibiotic pairings to phage-phage combinations within cocktails, highlighting the need for careful experimental evaluation (32).

Several mechanisms plausibly explain the observed antagonism. Competitive binding to bacterial surface receptors is a plausible mechanism, particularly given that four of the five selected phages belong to the *Phikmvirus* genus, with 88%–94.8% genomic similarity. Recent research emphasizes that effective phage cocktails should combine phages using non-redundant receptors to prevent competitive interference and suppress bacterial resistance (8, 27). When phages target overlapping lipopolysaccharide or outer membrane protein epitopes, adsorption rates decrease compared to single-phage applications (26). Studies have demonstrated that co-administration of phages can alter replication kinetics, with some phages experiencing reduced progeny production when competing for the same bacterial host (33). This receptor competition likely compromised the efficacy of the antagonistic cocktails, whose components may compete for identical binding sites.

Superinfection exclusion is another plausible mechanism, whereby initial phage infection activates bacterial defense systems, such as receptor modification, restriction-modification systems, or CRISPR-Cas immunity, which block subsequent infections (34). Antagonistic interactions can arise through diverse mechanisms, including reduced phage replication, disrupted counter-defenses, and altered bacterial surface structures (32, 35). The consistent underperformance of cocktails containing Phages 1 + 2 or 2 + 3 + 5 suggests specific incompatibilities, while all five antagonistic cocktails excluded Phage 4, indicating that Phage 4’s unique properties mitigate antagonism when present. These mechanisms are speculative and were not directly tested in this study; future work incorporating receptor mapping and adsorption kinetic measurements is warranted.

In contrast, Cocktail D (Phages 1 + 3 + 4) exhibited synergy. This rarity, as shown in this study, underscores the importance of precise phage selection. Multiple context-dependent factors influence phage interactions, and predicting clinical applicability requires careful experimental validation rather than relying on theoretical predictions (36). Cocktail D’s synergistic performance likely derives from complementary host recognition mechanisms. While Phages 1 and 3 are closely related, they belong to different *Phikmvirus* species, and Phage 4 shows moderate genomic divergence. This diversity enables targeting of distinct bacterial surface structures without competitive interference. Studies have shown that functionally diverse phage combinations targeting different adsorption receptors achieve higher efficacy at lower phage richness levels (27). Additionally, Phage 4’s extended inhibition duration (21.0 h) compared to Phages 1 and 3 (11–12 h) suggests temporal complementarity, where rapid initial killing is followed by prolonged suppression.

Multi-strain biofilm inhibition results critically address the single-strain limitation of initial screening. Cocktail D demonstrated consistent antibiofilm activity across eight strains with variable genetic backgrounds. The highest efficacy against MTPA35, which possesses a genetic profile with partial *pel* operon deletion but intact *psl* systems, suggests that reliance on a single exopolysaccharide system creates vulnerability to phage disruption. Phage cocktails must carefully consider potential antagonistic interactions between phages, as such interactions can compromise efficacy in biofilm eradication (37).

The *G. mellonella in vivo* validation provides evidence of therapeutic efficacy in a complex biological system. Recent studies have demonstrated that phage cocktails against various pathogens in *G. mellonella* models achieve survival rates of 60%–100%, particularly when treatment is administered prophylactically or shortly after infection (12, 38, 39). Preservation of activity scores, melanization scores, and cocoon formation indicates that phage treatment not only prevented mortality but also restored near-normal physiological function. The absence of toxicity in phage-only controls is particularly significant for clinical translation, as safety concerns regarding phage endotoxins and immune activation have historically limited therapeutic adoption (40).

This study has several limitations. Cocktail screening was conducted against a single reference strain, PA01, restricting the generalizability of interaction classifications to other strain backgrounds. Additionally, biofilm CFU enumeration relied on mechanical dispersal (vortexing), which may not achieve complete single-cell disaggregation; as a conservative limitation, phage-mediated EPS depolymerization may differentially enhance dispersibility in treated biofilms, potentially leading to slight overestimation of viable cell reduction. Several converging lines of evidence suggest that this artifact, while possible, is unlikely to account for the magnitude of the observed reductions. First, the dispersion protocol employed is consistent with widely adopted published standards for viable cell recovery from static polystyrene microplate biofilms, and any inherent dispersibility limitations apply equally across all experimental conditions. Second, the observed reductions of 2.7–3.6 log_10_ CFU substantially exceed the less-than-one-log recovery error typically attributable to incomplete disaggregation in comparable systems, rendering a dispersibility artifact an unlikely primary explanation for effects of this magnitude. Third, CFU reductions were concordant with crystal violet biomass measurements across all responsive isolates; because the crystal violet assay quantifies total biofilm biomass through dye binding and does not require cell disaggregation, it constitutes a mechanistically independent line of evidence supporting genuine biofilm disruption. Fourth, isolates resistant to the phage cocktail showed negligible CFU reduction despite undergoing the identical scraping and vortexing protocol; as phage-mediated EPS remodeling depends on active phage replication, resistant isolates would not be expected to undergo differential EPS degradation even in the presence of phage, and their lack of response is therefore consistent with a biological treatment effect rather than a protocol-driven dispersibility artifact. Taken together, these observations indicate that while differential disaggregation cannot be formally excluded as a contributing factor, it is unlikely to account for the magnitude, consistency, or isolate specificity of the reported CFU reductions. Finally, mechanistic explanations proposed for antagonistic and synergistic interactions (receptor competition and superinfection exclusion) are speculative and were not directly tested in this study; future work incorporating receptor mapping and adsorption kinetic measurements is warranted. Future studies should validate top-performing cocktails against panels of ≥30 strains representing diverse resistance mechanisms and receptor profiles.

### Conclusion

The predominance of antagonism (50%) over synergy (10%) in this study challenges prevailing assumptions in phage therapy. Synergistic and antagonistic interactions are highly dependent on the mechanism of action, with host factors potentially suppressing beneficial interactions (36). This study demonstrates that combining phages can reduce efficacy below the best individual component, emphasizing the need for experimental validation rather than the assumption of additive benefits. The HSA independence model provides a quantitative framework enabling standardized comparison across studies and data-driven identification of therapeutically superior combinations, advancing phage therapy beyond empirical formulation by quantifying phage interactions prior to *in vivo* commitment.

## Data Availability

The complete genome sequences for the five bacteriophages used in this study have been deposited in GenBank. Phage vB_PaePA01phi1_RS1 (Phage 1), vB_PaePA10145phi1_RS1 (Phage 2), vB_PaePA8132phi1_PS3 (Phage 3), vB_PaePA10145phi1_HR2 (Phage 4), and vB_PaePA10145phi1_MKR2 (Phage 5) are available under BioSample accession numbers SAMN47761291, SAMN47761292, SAMN47761293, SAMN47761294, and SAMN47761295, respectively. The corresponding GenBank accession numbers are PV565004, PV565005, PV565006, PV565007, and PV565008, and Sequence Read Archive (SRA) accession numbers are SRR33007766, SRR33007765, SRR33007764, SRR33007763, and SRR33007762, respectively. All sequences are associated with BioProject PRJNA1245669.

## SUPPLEMENTAL MATERIAL

Supplemental material spectrum.04096-25-s0001.docx Fig. S1 to S4; Tables S1 to S5.

ASM does not own the copyrights to Supplemental Material that may be linked to, or accessed through, an article. The authors have granted ASM a non-exclusive, world-wide license to publish the Supplemental Material files. Please contact the corresponding author directly for reuse.
