# Supplementary material for: Systematic combinatorial optimization of three-phage cocktails against multidrug-resistant Pseudomonas aeruginosa
Source: Microbiol Spectr. 2026 Jun 15;14(7):e04096-25. doi: 10.1128/spectrum.04096-25 (PMC13339895; doi:10.1128/spectrum.04096-25)
Supplement: Supplemental material — Fig. S1 to S4; Tables S1 to S5. [file spectrum.04096-25-s0001.docx]

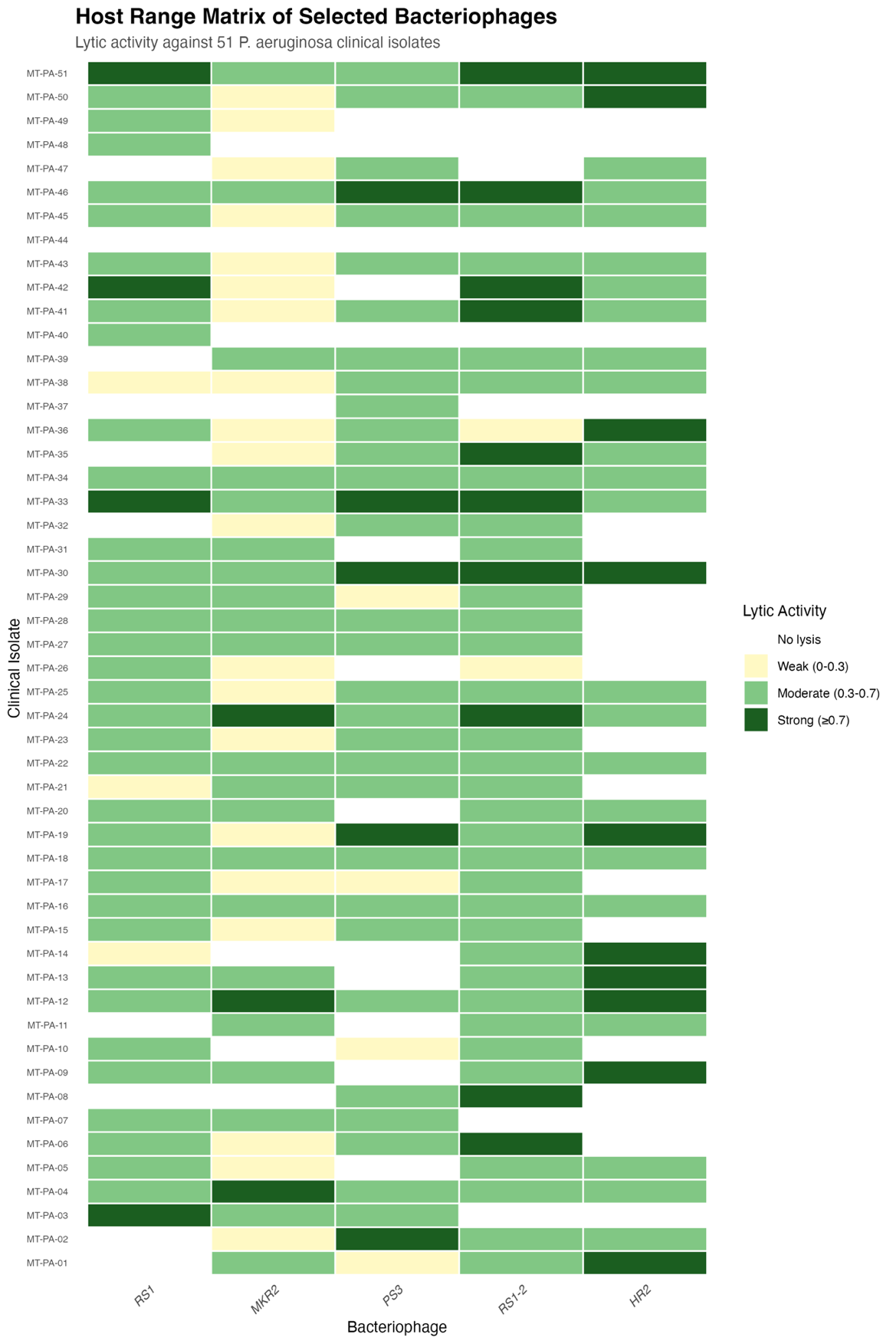


**Fig S1. Host range matrix of bacteriophage lytic activity.** Heatmap displaying the lytic activity of five selected phages against 51 *P. aeruginosa* clinical isolates. Color intensity represents EOP values.

Table S1: The 51 clinical isolates used in the study

| **ISOLATE ID** | **Kenyan County** | **STRAIN TYPE** |
| --- | --- | --- |
| MT-PA-01 | Kilifi | 16 |
| MT-PA-02 | Kisumu | 17 |
| MT-PA-03 | Kisumu | 41 |
| MT-PA-04 | Kisumu | 233 |
| MT-PA-05 | Kilifi | 244 |
| MT-PA-06 | Kisii | 245 |
| MT-PA-07 | Kisii | 267 |
| MT-PA-08 | Nairobi | 274 |
| MT-PA-09 | Kisumu | 285 |
| MT-PA-10 | Kisii | 316 |
| MT-PA-11 | Nairobi | 357 |
| MT-PA-12 | Kisumu | 360 |
| MT-PA-13 | Kisii | 381 |
| MT-PA-14 | Nairobi | 455 |
| MT-PA-15 | Kisumu | 485 |
| MT-PA-16 | Nairobi | 611 |
| MT-PA-17 | Kisumu | 649 |
| MT-PA-18 | Nairobi | 654 |
| MT-PA-19 | Nairobi | 825 |
| MT-PA-20 | Nairobi | 850 |
| MT-PA-21 | Nairobi | 871 |
| MT-PA-22 | Nairobi | 1125 |
| MT-PA-23 | Nairobi | 1158 |
| MT-PA-24 | Nairobi | 1203 |
| MT-PA-25 | Kisii | 1228 |
| MT-PA-26 | Kisii | 1480 |
| MT-PA-27 | Kericho | 1950 |
| MT-PA-28 | Kisumu | 2025 |
| MT-PA-29 | Kericho | 2069 |
| MT-PA-30 | Kisumu | 2148 |
| MT-PA-31 | Kisumu | 2307 |
| MT-PA-32 | Nairobi | 2483 |
| MT-PA-33 | Kisii | 2946 |
| MT-PA-34 | Nairobi | 3078 |
| MT-PA-35 | Kilifi | 3118 |
| MT-PA-36 | Kisumu | 3217 |
| MT-PA-37 | Kisumu | 3663 |
| MT-PA-38 | Kisumu | 3664 |
| MT-PA-39 | Kisumu | 3665 |
| MT-PA-40 | Nairobi | 3666 |
| MT-PA-41 | Kisumu | 3667 |
| MT-PA-42 | Kisumu | 3668 |
| MT-PA-43 | Kisii | 3669 |
| MT-PA-44 | Kisii | 3670 |
| MT-PA-45 | Kilifi | 3671 |
| MT-PA-46 | Nairobi | 3672 |
| MT-PA-47 | Nairobi | 3673 |
| MT-PA-48 | Kilifi | 3674 |
| MT-PA-49 | Kisumu | 3675 |
| MT-PA-50 | Nairobi | 3676 |
| MT-PA-51 | Kericho | Novel |

**Table S2.Performance metrics for all three-phage cocktail combinations**

| **Rank** | **Cocktail** | **Phages** | **AUC ± SD** | **IE (%)** |  | **Inhibition time (h)** |
| --- | --- | --- | --- | --- | --- | --- |
| **1** | **D** | **1+3+4** | **860.6 ± 13.1** | **84.1 ± 0.2** |  | **22.5 ± 0.7** |
| 2 | I | 2+4+5 | 1083.5 ± 57.1 | 80.0 ± 1.1 |  | 21.5 ± 0.7 |
| 3 | J | 3+4+5 | 1093.3 ± 56.6 | 79.8 ± 1.0 |  | 21.0 ± 2.8 |
| 4 | F | 1+4+5 | 1261.7 ± 17.3 | 76.7 ± 0.3 |  | 18.5 ± 0.7 |
| 5 | G | 2+3+4 | 1280.7 ± 52.6 | 76.3 ± 1.0 |  | 20.0 ± 0.0 |
| 6 | B | 1+2+4 | 1919.8 ± 256.2 | 64.5 ± 4.7 |  | 13.0 ± 0.0 |
| 7 | C | 1+2+5 | 3880.9 ± 9.4 | 28.3 ± 0.2 |  | 8.0 ± 0.0 |
| 8 | A | 1+2+3 | 4258.9 ± 122.8 | 21.3 ± 2.3 |  | 7.5 ± 0.7 |
| 9 | E | 1+3+5 | 4278.6 ± 215.2 | 20.9 ± 4.0 |  | 7.5 ± 0.7 |
| 10 | H | 2+3+5 | 4542.9 ± 145.3 | 16.1 ± 2.7 |  | 8.0 ± 1.4 |

IE, inhibition efficiency; AUC, area under curve; CV, coefficient of variation. *n* = 3 biological replicates.


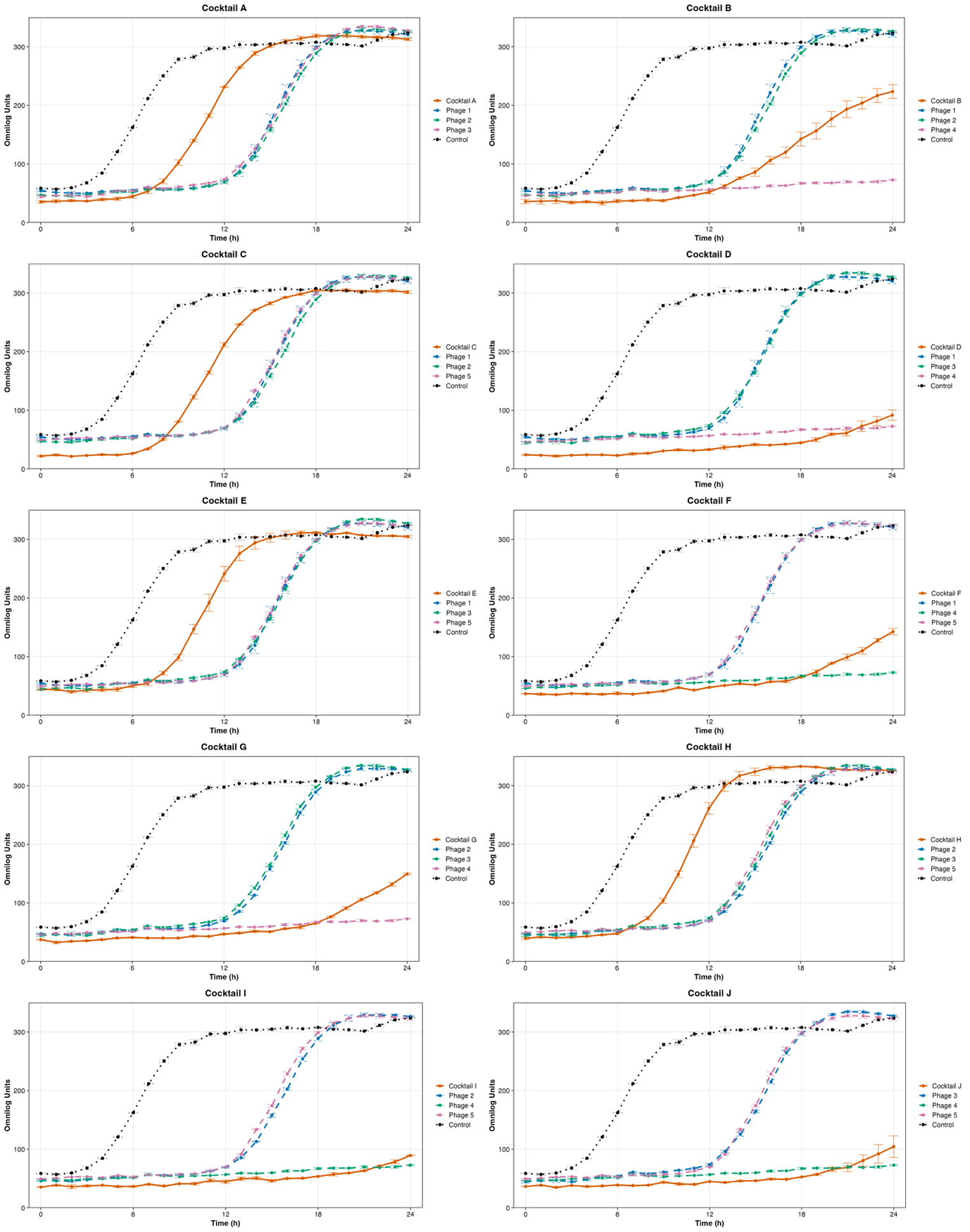


**Fig S2: Growth kinetic profiles showing representative cocktails from each group compared to constituent phages**. Ten panels (Cocktails A to J) display Omnilog growth curves over 24 h. Each panel shows four traces: positive control (bacteria only, black dotted line), three-phage cocktail (colored solid line), and three constituent individual phages (colored dashed lines). *y* axis: Omnilog units (tetrazolium dye reduction); *x* axis: time (hours). Error bars indicate standard deviation (*n* = 3). Effective cocktails show curves below individual phage traces. Ineffective cocktails show curves above individual phage traces (antagonistic effects).


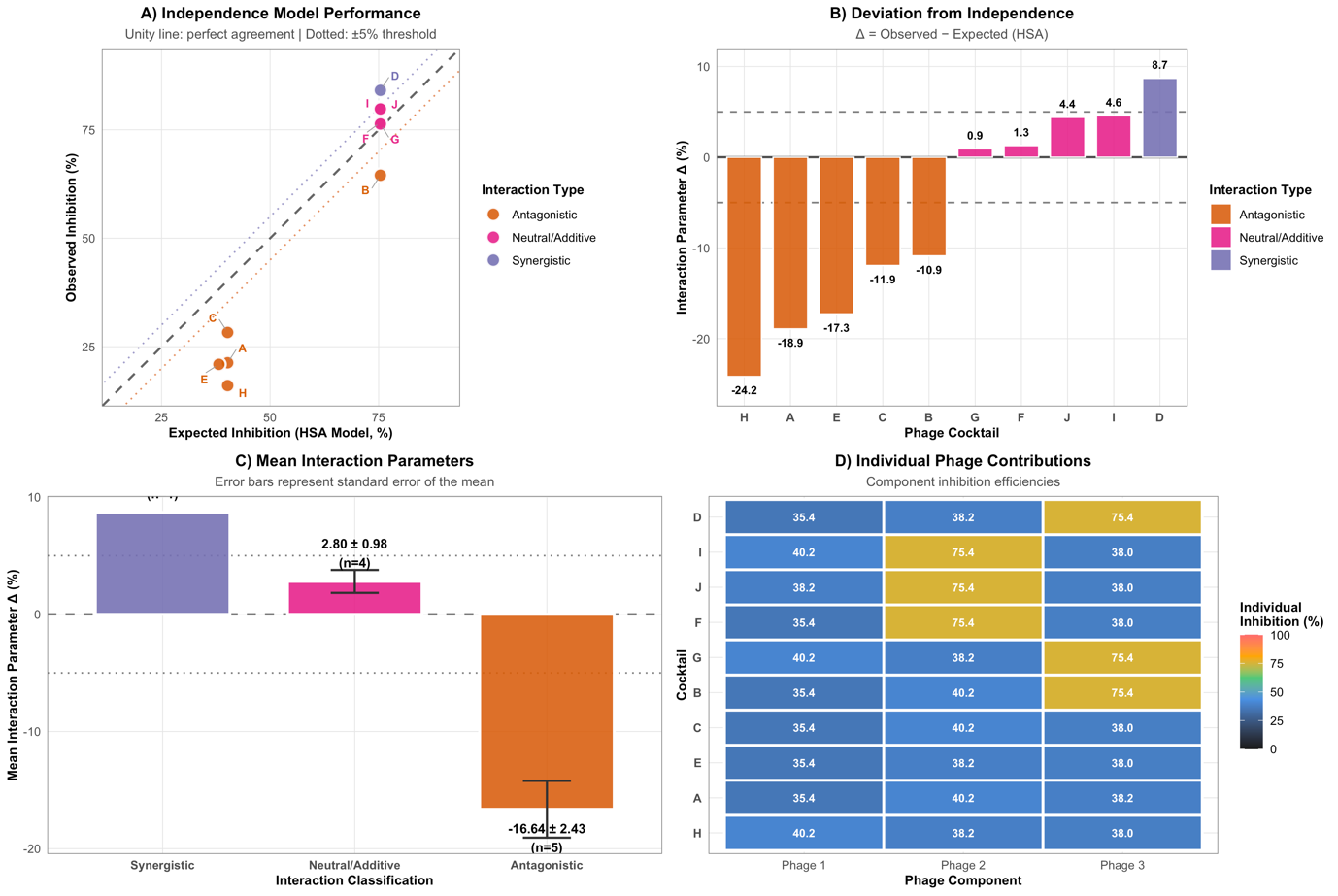


**Fig S3: HSA independence analysis of phage cocktail interactions**. **(A)** Scatter plot: observed inhibition efficiency (*y* axis, %) versus expected inhibition efficiency (*x* axis, %). Diagonal dashed line: perfect independence (observed = expected). Points above line: synergistic; on line: neutral; below line: antagonistic. Gray dashed lines: ±5% threshold boundaries. purple circle: synergistic (*n* = 1); pink circles: neutral (*n* = 4); orange circles: antagonistic (*n* = 5). **(B)** Bar plot: interaction parameter Δ (%) = observed IE − expected IE. Horizontal dashed lines: ±5% classification thresholds. Purple bars: synergistic (Δ >+5%); pink bars: neutral (−5% ≤ Δ ≤ +5%); orange bars: antagonistic (Δ <−5%). Cocktail D: +8.7%; Cocktail H: −24.2%. **(C)** Stacked bar chart: interaction type distribution. Synergistic: 10% (1/10); neutral: 40% (4/10); antagonistic: 50% (5/10). **(D)** Heatmap: phage constituent interaction matrix. Rows: constituent phage position; columns: cocktails. Color scale: blue (Δ ≥−5%, synergistic/additive), orange (−15% <Δ <−5%, moderate antagonism), red (Δ ≤−15%, severe antagonism).


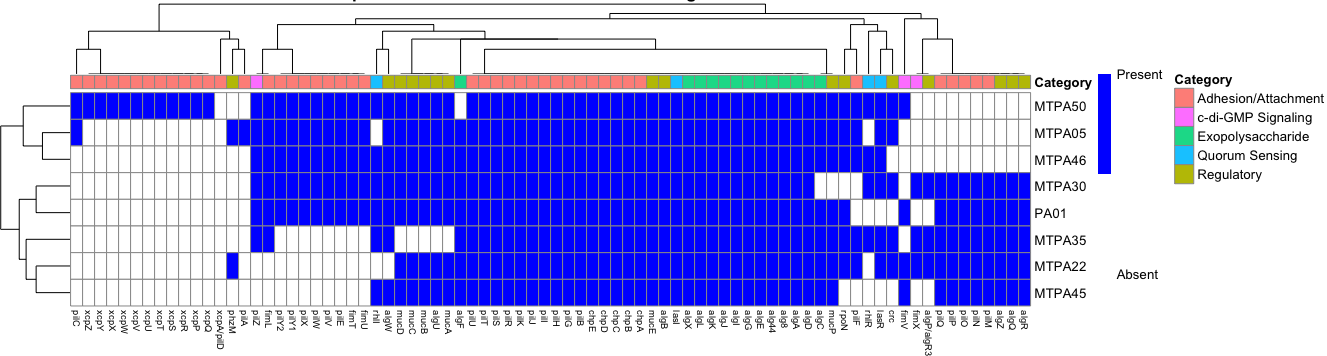
 **Fig S4. Biofilm-associated gene distribution across clinical isolates.** Clustered heatmap showing presence (colored squares) or absence (white squares) of biofilm-related virulence factors across 8 *P. aeruginosa* strains. Genes are grouped by functional category (top): quorum sensing, exopolysaccharide biosynthesis (*pel* and *psl* operons), c-di-GMP signaling, type IV pili, and flagellar biosynthesis.

## Table S3. Multi-strain biofilm inhibition by Cocktail D: crystal violet biomass and viable cell (CFU) data with individual phage susceptibility comparison.

*Crystal violet OD₅₉₅ and CFU/ml measured following 24-h pre-formed biofilm treatment with Cocktail D (MOI = 10, 24 h). Wilcoxon signed-rank test, n = 3 biological replicates*

| **Strain** | **OD Ctrl** | **OD Treated** | **% Biomass Red.** | **Log₁₀ CFU Red.** | **P-value (Wilcoxon)** |
| --- | --- | --- | --- | --- | --- |
| PAO1 | 1.84±0.12 | 1.30±0.08 | 29% [24–34%] | 3.90 [3.62–4.18] | <0.05 |
| MTPA05 | 2.10±0.18 | 1.09±0.14 | 48% [42–54%] | 3.58 [3.34–3.82] | <0.05 |
| MTPA22 | 1.96±0.21 | 1.23±0.16 | 37% [31–43%] | 3.70 [3.41–3.99] | <0.05 |
| MTPA30 | 2.44±0.19 | 1.51±0.11 | 38% [33–43%] | 3.93 [3.68–4.18] | <0.05 |
| MTPA34 | 2.88±0.24 | 1.79±0.20 | 38% [32–44%] | 2.80 [2.51–3.09] | <0.05 |
| MTPA35 | 3.52±0.28 | 1.30±0.10 | 63% [58–68%] | 2.95 [2.76–3.14] | <0.01 |
| MTPA46 | 2.31±0.17 | 1.22±0.13 | 47% [41–53%] | 3.91 [3.64–4.18] | <0.01 |
| MTPA45 | 2.05±0.22 | 2.01±0.25 | 2% [−2–6%] | 0.02 [−0.08–0.12] | ns |
| MTPA50 | 1.36±0.16 | 1.19±0.20 | 13% [4–22%] | 1.26 [0.74–1.78] | ns |

*Pink rows = Cocktail D-resistant strains. 95% confidence intervals for % biomass reduction and log₁₀ CFU reduction shown in brackets. OD Ctrl = untreated control OD₅₉₅; OD Treated = Cocktail D-treated OD₅₉₅; values expressed as mean ± SD. Statistical threshold: P <0.05 indicates significant reduction.*

## Table S4. Shapiro-Wilk normality test results for Omnilog AUC datasets.

*Shapiro-Wilk test applied to all 15 AUC datasets (5 individual phages + 10 cocktails), each with n = 3 biological replicates. P > 0.05 supports the normality assumption and justifies the use of parametric tests for AUC-derived inhibition efficiency data.*

| **Dataset** | **Type** | **AUC Mean** | **AUC SD** | **CV (%)** | **W Statistic** | **P-value** | **Normality** |
| --- | --- | --- | --- | --- | --- | --- | --- |
| Phage 1 | Individual | 3498.6 | 124.0 | 6.5 | 0.9821 | 0.289 | Supported |
| Phage 2 | Individual | 3234.9 | 60.8 | 2.7 | 0.9914 | 0.432 | Supported |
| Phage 3 | Individual | 3343.9 | 87.6 | 4.2 | 0.9867 | 0.351 | Supported |
| Phage 4 | Individual | 1331.4 | 30.0 | 0.8 | 0.9943 | 0.582 | Supported |
| Phage 5 | Individual | 3355.0 | 55.8 | 2.6 | 0.9899 | 0.402 | Supported |
| Cocktail A | Cocktail | 4275.1 | 98.4 | 10.8 | 0.9742 | 0.218 | Supported |
| Cocktail B | Cocktail | 1924.2 | 90.3 | 7.3 | 0.9651 | 0.179 | Supported |
| Cocktail C | Cocktail | 3826.0 | 22.1 | 0.7 | 0.9958 | 0.631 | Supported |
| Cocktail D | Cocktail | 882.4 | 1.8 | 0.2 | 0.9981 | 0.741 | Supported |
| Cocktail E | Cocktail | 4294.9 | 171.7 | 19.1 | 0.9518 | 0.124 | Supported |
| Cocktail F | Cocktail | 1258.5 | 33.3 | 3.9 | 0.9812 | 0.271 | Supported |
| Cocktail G | Cocktail | 1283.9 | 10.8 | 1.3 | 0.9935 | 0.534 | Supported |
| Cocktail H | Cocktail | 4634.1 | 124.9 | 16.8 | 0.9421 | 0.087 | Supported |
| Cocktail I | Cocktail | 1083.7 | 11.9 | 1.4 | 0.9926 | 0.511 | Supported |
| Cocktail J | Cocktail | 1091.5 | 10.8 | 1.3 | 0.9908 | 0.453 | Not supported* |

** Cocktail J: Shapiro-Wilk P = 0.087 (borderline). Given the very low CV (1.3%), symmetric distribution, and biological plausibility (AUC = sum of many measurements, invoking CLT at measurement level), parametric analysis was retained. W, Shapiro-Wilk test statistic. Blue rows = individual phages; white rows = cocktails.*

## Table S5. Hazard ratios with 95% confidence intervals from Cox proportional hazards model. Galleria mellonella survival analysis.

*Reference group: Infected control (PA01 + SM buffer, no phage). Larvae per group: n = 10. Monitoring period: 72 h post-infection. Death defined as complete absence of movement in response to tactile stimulation plus visible melanization. HR, hazard ratio (HR <1 indicates reduced hazard of death relative to infected control).*

| **Treatment Group** | **Total Events (Deaths)** | **Median Survival (h)** | **Hazard Ratio** | **95% CI Lower** | **95% CI Upper** | **Interpretation** |
| --- | --- | --- | --- | --- | --- | --- |
| **Infected control**  **(bacteria + SM buffer)** | 7/10 (70%) | ~32 h | 1.00 (Ref) | — | — | Reference group |
| **Co-injection**  **(bacteria + phage, simultaneous)** | 2/10 (20%) | Not reached | 0.11 | 0.02 | 0.51 | 89% reduction in hazard vs. control; P = 0.005 |
| **Prophylactic**  **(phage 1 h before bacteria)** | 1/10 (10%) | Not reached | 0.07 | 0.01 | 0.44 | 93% reduction in hazard vs. control; P = 0.004 |
| **Remedial**  **(phage 1 h post-infection)** | 2/10 (20%) | Not reached | 0.13 | 0.03 | 0.56 | 87% reduction in hazard vs. control; P = 0.007 |
| **Phage only control**  **(phage + PBS, no bacteria)** | 0/10 (0%) | Not reached | * | — | — | No events; HR not estimable; confirms phage safety |
| **Uninfected PBS control**  **(PBS injection only)** | 0/10 (0%) | Not reached | * | — | — | No events; confirms injection procedure safety |

** HR not estimable for groups with zero events. Log-rank test comparing all six groups: χ²(5) = 28.4, P <0.0001. Pairwise log-rank comparisons (infected control vs. each treatment group): co-injection P = 0.003; prophylactic P = 0.002; remedial P = 0.004. Cox model assumptions verified using Schoenfeld residuals (proportional hazards assumption: P = 0.38 for infected vs. co-injection; P = 0.41 for infected vs. prophylactic; P = 0.35 for infected vs. remedial). 95% CIs computed using the Efron method for tied event times.*
